# Immune Dysfunctions of CD56^neg^ NK Cells Are Associated With HIV-1 Disease Progression

**DOI:** 10.3389/fimmu.2021.811091

**Published:** 2022-01-07

**Authors:** Wen-Jing Cao, Xiao-Chang Zhang, Lin-Yu Wan, Qing-Yu Li, Xiu-Ying Mu, An-Liang Guo, Ming-Ju Zhou, Li-Li Shen, Chao Zhang, Xing Fan, Yan-Mei Jiao, Ruo-Nan Xu, Chun-Bao Zhou, Jin-Hong Yuan, Sheng-Qi Wang, Fu-Sheng Wang, Jin-Wen Song

**Affiliations:** ^1^ The First Affiliated Hospital of USTC, Division of Life Sciences and Medicine, University of Science and Technology of China, Hefei, China; ^2^ Beijing Institute of Radiation Medicine, Beijing, China; ^3^ Department of Clinical Medicine, Bengbu Medical College, Bengbu, China; ^4^ Department of Infectious Diseases, the Fifth Medical Center of Chinese PLA General Hospital, National Clinical Research Center for Infectious Diseases, Beijing, China

**Keywords:** HIV-1, CD56^neg^ NK cells, scRNA-seq, CD39, dysfunction

## Abstract

**Background:**

Populations of natural killer cells lacking CD56 expression [CD56^neg^ natural killer (NK) cells] have been demonstrated to expand during human immunodeficiency virus (HIV)-1 infection. However, their phenotypic and functional characteristics have not been systematically analyzed, and their roles during disease progression remain poorly understood.

**Methods:**

In this study, 84 donors, namely 34 treatment-naïve HIV-1-infected patients (TNs), 29 HIV-1-infected patients with successful antiretroviral therapy (ARTs), and 21 healthy controls (HCs), were enrolled. The phenotypic and functional characteristics of CD56^neg^ NK cells were analyzed using single-cell RNA-sequencing (scRNA-seq) and flow cytometry. A potential link between the characteristics of CD56^neg^ NK cells and the clinical parameters associated with HIV-1 disease progression was examined.

**Results:**

The frequency of the CD56^neg^ NK cell population was significantly increased in TNs, which could be partially rescued by ART. Flow cytometry analyses revealed that CD56^neg^ NK cells were characterized by high expression of CD39, TIGIT, CD95, and Ki67 compared to CD56^dim^ NK cells. *In vitro* assays revealed reduced IFN-γ and TNF-α secretion, as well as decreased expression of granzyme B and perforin in CD56^neg^ NK cells. In line with the data obtained by flow cytometry, scRNA-seq analysis further demonstrated impaired cytotoxic activities of CD56^neg^ NK cells. Notably, a negative correlation was observed between CD39, CD95, and Ki67 expression levels in CD56^neg^ NK cells and CD4^+^ T cell counts.

**Conclusions:**

The results presented in this study indicate that the CD56^neg^ NK cell population expanded in HIV-1-infected individuals is dysfunctional and closely correlates with HIV-1 disease progression.

## Introduction

Natural killer (NK) cells are crucial components of the innate immune system and play a key role in the first-line of defense against tumor cells and viral infections (1–3). Regulation of NK cell effector functions is ensured by a dynamic equilibrium between inhibitory and activating cell surface receptors (4, 5). In healthy adults, NK cells constitute about 5–15% of peripheral blood mononuclear cells (PBMCs), and three major NK cell subsets can be identified based on the expression of CD56 and CD16 surface markers: CD56^bright^CD16^-^ (CD56^bri^) NK cells, which are efficient producers of cytokines (6); CD56^+^CD16^+^ (CD56^dim^) NK cells, which represent the predominant NK cell subset and display cytolytic functions by establishing direct contacts or *via* antibody-dependent cell-mediated cytotoxicity (7); and CD56^-^CD16^+^ (CD56^neg^) NK cells, which have been shown to expand during viral infections to form an “anergic” population. However, the CD56^neg^ subset has not been studied in-depth. Thus, further research is needed to provide a better understanding of the immunological characteristics of CD56^neg^ NK cells.

NK cells are immune effectors, the functions of which are closely linked to human immunodeficiency virus (HIV)-1 disease progression. It has been reported that during HIV-1 infection, the activating receptor-encoding KIR allele *KIR3DS1*, in combination with HLA-B alleles encoding an isoleucine at position 80 (*HLA-B Bw4-80Ile*), was associated with delayed progression to AIDS (8). In humanized mice, ALT-803, an IL-15 superagonist, could activate NK cells *in vivo* to potently suppress acute HIV-1 infection (9). Through single-cell RNA-sequencing (scRNA-seq) of PBMCs during an acute HIV-1 infection, Kazer et al. found that two participants who maintained viral loads < 1000 copies mL^-1^ at 2.75 years after infection without antiretroviral therapy (ART) exhibited an increase in proliferative and cytotoxic NK cells during the early stages of infection (10). In addition, inducible expression of NKp46 and NKp30, as well as interferon gamma (IFN-γ) production upon NK cell activation were shown to correlate inversely with the size of the HIV-1 DNA reservoir (11). Moreover, NK cells from elite controllers (EC) and long-term nonprogressors displayed increased NKG2D expression, significant upregulation of HLA-DR, and increased CD57 expression (12). Thus, NK cells constitute promising candidates for the development of a functional cure for HIV-1.

Numerous studies have been carried out to characterize the phenotypic and functional changes in NK cells during HIV-1 infection (13, 14). For example, expression of major triggering receptors, such as NKp30, NKp46, and NKp44, was found to be decreased in chronic HIV-1 infection (15, 16), whereas expression of inhibitory receptors, such as T cell immunoreceptor with Ig and ITIM domains (TIGIT), was increased (17, 18), leading to impaired cytotoxic NK cell functions. It has been reported that the unresponsiveness and impaired killing activity of NK cells resulted from a downregulation of activating receptors and an upregulation of inhibitory receptors during HIV-1 infection (19, 20). In 1995, unconventional CD56^neg^ NK cell populations were first discovered and demonstrated to be increased in HIV-1-infected patients (21, 22). Subsequently, Alter et al. reported that an increase in CD56^neg^ NK cell numbers was associated with a high HIV-1 viral load (23). In addition, there was no significant change in the proportion of CD56^neg^ NK cells at the early stage, but a significant increase during the chronic stage of HIV-1 infection, suggesting that sustained viral replication may be related to the accumulation of CD56^neg^ NK cells (24, 25). Furthermore, the percentage of IL-10- and TGF-β-producing CD56^neg^ NK cells was higher than that of CD56^dim^ NK cells. These CD56^neg^ NK cells could inhibit IFN-γ production by autologous CD8^+^ T cells (26). Collectively, these lines of evidence suggest that the accumulation of CD56^neg^ NK cells in patients with chronic HIV-1 infection is significant. However, the relationship between CD56^neg^ NK cell characteristics and HIV-1 disease progression is not well understood.

In this study, the characteristics of CD56^neg^ NK cells from HIV-1-infected individuals were analyzed using flow cytometry and scRNA-seq. Our findings revealed that CD56^neg^ NK cell populations expanded during chronic HIV-1 infection displayed impaired cytotoxicity and reduced cytokine production. These CD56^neg^ NK cell-related immune dysfunctions correlated with HIV-1 disease progression.

## Materials and Methods

### Study Participants

A total of 63 HIV-1-infected patients and 21 healthy controls (HCs) were recruited from the Fifth Medical Center of Chinese PLA General Hospital, Beijing, China. Infected patients comprised 34 treatment-naïve HIV-1-infected patients (TNs) who exhibited typical progressive disease and did not receive ART and 29 HIV-1-infected patients who received successful ART (ARTs) for more than one year, with plasma HIV-1 RNA levels below the detection limit (**Table 1** and **Supplementary Table 1**). Exclusion criteria included coinfection with hepatitis B (HBV) or C virus (HCV), pregnancy, and a moribund status.

**Table 1:** Clinical characteristics of study participants.

	HCs (n = 21)	TNs (n = 34)	ARTs (n = 29)
Age, years, median (IQR)	29 (28~34)	32 (25~38)	32 (29~36)
Gender, male/female	11/10	34/0	29/0
Plasma level of HIV-1 RNA, Log copies/mL, median (IQR)	NA	3.74 (3.19~4.18)	<LDL
CD4 count, cells/μL, median (IQR)	783 (594~905)	358 (309~449)	516 (462~670)
CD8 count, cells/μL, median (IQR)	595 (520~661)	1010.5 (660~1376)	736 (464~939)
CD4/CD8 ratio, median (IQR)	1.27 (1.05~1.49)	0.33 (0.22~0.53)	0.83 (0.67~1.09)

IQR, interquartile range; HCs, healthy controls; TNs, treatment-naïve HIV-1-infected patients; ARTs, HIV-1-infected patients with successful antiretroviral therapy (ART); n, number of individuals per group; NA, not applicable; LDL, low detection limit.

### PBMC Isolation

PBMCs were isolated from EDTA anti-coagulated venous blood by Ficoll-Hypaque (MD Pacific Biotechnology, Tianjin, China) density gradient centrifugation. All blood samples were collected with the approval of the Fifth Medical Center of Chinese PLA General Hospital Research Ethics Committee. The study subjects provided written informed consent to participate in this study, which was in accordance with the principles laid down in the Declaration of Helsinki.

### Detection of Plasma HIV-1 RNA

The HIV-1 RT-PCR Fluorescence Quantitative Detection Kit (Bioer Technology, Hangzhou, China) was used according to the manufacturer’s instructions to quantify HIV-1 RNA levels in plasma. PCR reactions were performed using a CFX96 real-time polymerase chain reaction system (Bio-Rad, Hercules, CA, USA).

### Flow Cytometry

For phenotypic staining, PBMCs were stained extracellularly by 30 min of incubation with primary antibodies specific to the respective makers at 4°C in the dark. Fluorescently conjugated monoclonal antibodies and reagents were as follows: anti-CD3, anti-CD14, anti-CD20, anti-CD16, anti-CD56, anti-CD39, anti-CD62L, anti-NKp30, anti-NKp44, anti-NKp46, anti-NKG2A, anti-Tim-3, anti-CD69, anti-NKG2C, anti-CD57, anti-CCR7, anti-CD32, anti-CD95, anti-PD-1, anti-TIGIT, anti-CD7, and anti-NKG2D. For subsequent staining of intracellular markers, the cells were permeabilized using the Transcription Factor Staining Buffer Set (Thermo Fisher Scientific, USA), followed by incubation with the indicated antibodies (anti-Ki67, anti-EOMES, and anti-T-bet). For functional tests, PBMCs were cultured in RPMI 1640 medium containing 10% fetal calf serum (Gibco, USA) and stimulated with IL-12 (10 ng/mL; PeproTech, USA), IL-15 (10 ng/mL; BioLegend, USA), and IL-18 (50 ng/mL; BioLegend, USA) for 20 h (27). To analyze cytokine production by stimulated cells, GolgiStop (BD Biosciences, USA) and CD107a were added 5 h before cell harvest. Afterwards, the cells were harvested for detection of intracellular IFN-γ, TNF-α, granzyme B, and perforin. Specific antibodies used for staining were employed as described in **Supplementary Table 2**.

### Data Source and Processing of scRNA-seq Data

A previously obtained scRNA-seq dataset was downloaded from the National Center for Biotechnology Information, U.S. National Library of Medicine (https://www.ncbi.nlm.nih.gov/sra/?term=SRP150325) (28). This dataset included three individual datasets corresponding to the three subsets of NK cells (CD56^bri^, CD56^dim^, and CD56^neg^). They were imported to CellRanger (version 5.0.1) to map the reads against the human reference genome 38 (GRCh38). Barcodes and unique molecular identifiers (UMIs) were counted for each cell, and unexpressed genes were filtered out. Then, three gene expression matrices were obtained for each NK cell subset, which were integrated using R tools. A stricter quality control was conducted, and the cells were filtered according to the following criteria: (1) number of genes > 500; (2) number of UMIs > 800; and (3) percentage of mitochondria-expressed genes < 5%. Cells that did not meet the afore mentioned criteria were excluded from subsequent analyses. Next, the data were normalized for sequencing depth employing the “LogNormalize” method, and the 2,000 most strongly varying genes were selected for further analyses. After scaling the gene expression data, principal component analysis was performed using the 2,000 hits described above. To perform data visualization, the top 20 principal components were selected for dimensionality reduction using the t-distributed Stochastic Neighbor Embedding (*t*-SNE) method. To ensure all the cells for further analysis were NK cells, some extra data cleaning processes were performed. Firstly, these cells were clustered by the ‘FindClusters’ function with the parameter “resolution=1”. Then, the obtained clusters were annotated and these clusters expressing the markers including CD3D (T cell), MS4A1 (B cell) and LYZ (monocyte) were removed. Analyses were performed under R (v.3.6.0) with Seurat (v3.2.3) packages.

### Pseudotime Analysis

In order to predict the differentiation relationship among the CD56^bri^, CD56^dim^ and CD56^neg^ NK cells, pseudotime analysis was performed by monocle2 (29). The 2,000 most strongly varying features were selected to order cells. The pseudotime trajectory was plot by the function ‘plot_cell_trajectory’. Significantly changed genes along the pseudotime trajectory were identified using the ‘differentialGeneTest’ function and the top 100 genes were clustered into 5 clusters and displayed using the ‘plot_pseudotime_heatmap’ function.

### Identification of Differentially Expressed Genes (DEGs) and Gene Ontology (GO) Enrichment

To identify the DEGs across the three NK cell subsets, the “FindMarker” function of the Seurat package was employed using multiple threshold parameters, including an average log2 fold change ≥ 0.5, with a Benjamini-Hochberg-corrected *p* value ≤ 0.05, as well as detection in ≥ 10% of cells in at least one subtype. Obtained DEGs served as input for the “enrichGO” function in the clusterProfiler package for “biological process (BP)” enrichment analysis. Genome-wide annotation was performed the org.Hs.eg.db (v.3.10.0) package as annotation database. The top 15 most significantly enriched BP functions for both up- and downregulated DEGs were separately selected to be displayed by barplots (organized by gene count).

### Calculation of Function Module Scores for Each Cell

We used function module scores to compare the differences in specific cell states or functions between the three types of NK cells. These scores were calculated by the “AddModuleScore” function in the Seurat R package. Genes categorized into “cytotoxicity”, “cytokine and chemokine receptors”, “adhesion molecules”, and “exhaustion” function modules have been reported by others (30, 31). Genes involved in “apoptosis” were selected from the GO term APOPTOTIC SIGNALING PATHWAY (GO:0097190). The details of these function modules are listed in **Supplementary Table 3**.

### Enzyme-Linked Immunosorbent Assay (ELISA)

Concentrations of sCD14, sCD163, and TGF-β1 were measured in patient-derived frozen plasma specimens stored at -80°C using ELISA kits (R&D systems, Minneapolis, USA) according to the manufacturer’s instructions.

### Statistical Analysis

Statistical analysis was performed using GraphPad Prism software (version 8.0; GraphPad Software, San Diego, CA, USA). Continuous measurements are displayed as median (interquartile range, IQR) and categorical variables are expressed as count (%). Mann-Whitney U-tests were used for comparisons between two groups, and the Wilcoxon signed-rank test was used for matched pairs. Correlations between two quantitative variables were evaluated using Pearson’s rank correlation test. Statistical significance was set at a *p* < 0.05.

## Results

### Accumulation of CD56^neg^ NK Cells in Patients With HIV-1 Infection

**Figure 1:**
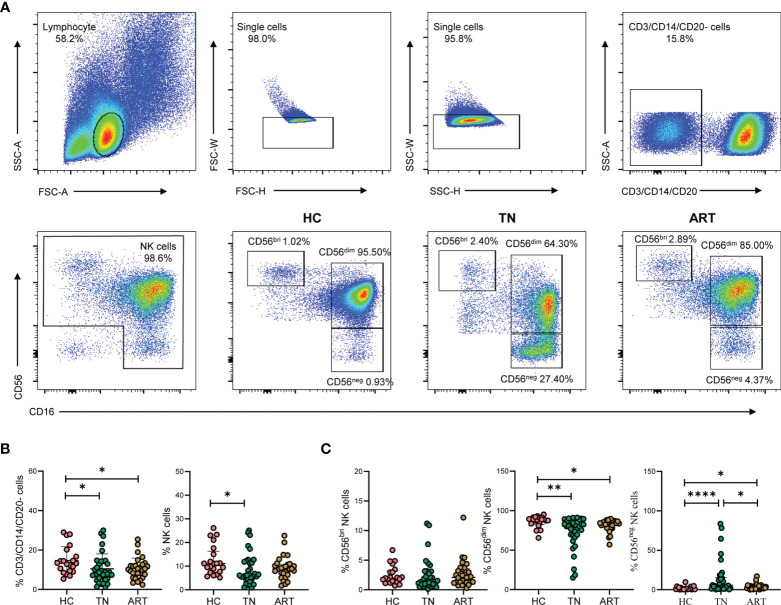
CD56^neg^ NK cells are expanded in peripheral blood of HIV-1-infected participants. **(A)** Gating strategy for flow cytometry analyses. NK cells were defined as negative for CD3, CD14, and CD20, but positive for either CD56 or CD16. NK cell subsets were further identified based on CD56 and CD16 expression on cells derived from healthy controls (HCs) and HIV-1-infected participants. The numbers indicate the percentages of cells within the gates. **(B, C)** HIV-1-infected participants included treatment-naïve HIV-1-infected patients (TNs) and HIV-1-infected patients with successful antiretroviral therapy (ARTs). Comparisons of the percentages of CD3/CD14/CD20^-^ cells, total NK cells **(B)** and the three NK cell subsets, namely CD56^bri^, CD56^dim^, and CD6^neg^ NK cells **(C)** in HCs (n = 21), TNs (n = 34), and ARTs (n = 29). Each dot represents one participant. Statistical significance between two groups was determined by Mann-Whitney U-test. *p < 0.05, **p < 0.01, and ****p < 0.0001.

To investigate the effect of HIV-1 infection on the proportions of NK cell subsets, three groups of individuals were included in this study: HCs (n = 17), TNs (n = 34), and ARTs (n = 29). Our gating strategy excluded CD3^+^ T cells, CD14^+^ monocytes, and CD20^+^ B cells. Three subsets of NK cells were identified based on CD56 and CD16 expression (**Figure 1A**). Compared with HCs, the frequencies of CD3/CD14/CD20^-^ cells and NK cells in PBMCs were decreased in the TNs (**Figure 1B**). In addition, the frequency of CD56^dim^ NK cells in total NK cells was also decreased compared to that observed in HCs. This was particularly pronounced in TNs (72.72% vs 85.72%, *p* = 0.0034). In contrast, the CD56^neg^ NK cell population was significantly expanded in TNs, but ART could partially restore the frequency of CD56^neg^ NK cells (**Figure 1C**). Taken together, these data indicate that CD56^neg^ NK cells expanded at the expense of a decrease in CD56^dim^ NK cells upon HIV-1 infection, which could not be fully rescued by ART.

### scRNA-seq-Based Characterization of CD56^neg^ NK Cells in an HIV-1-Infected Individual

**Figure 2:**
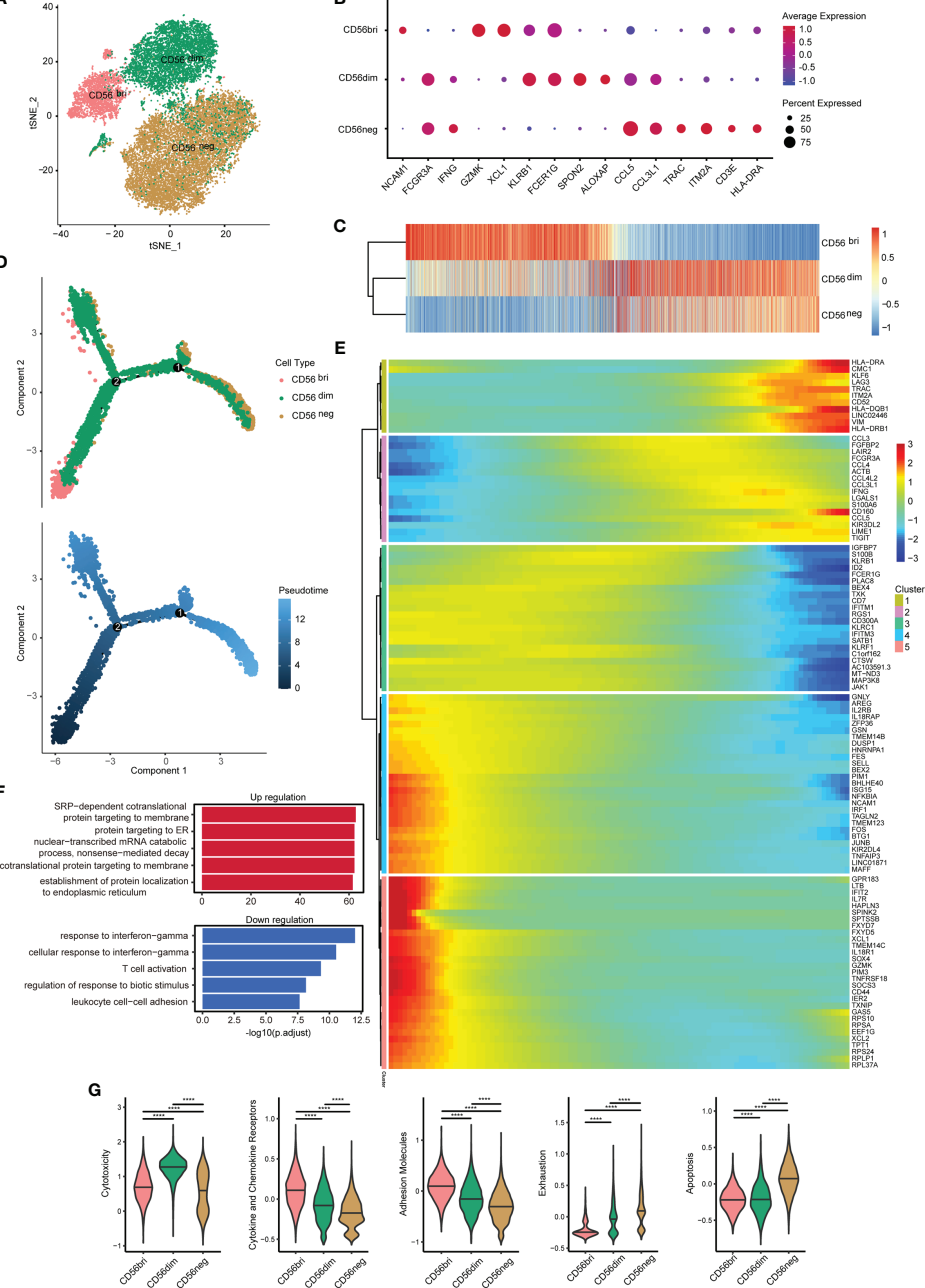
Single-cell gene expression analysis using peripheral blood from HIV-1-infected patients shows lower cytotoxicity, but higher exhaustion of CD56^neg^ NK cells. **(A)** *t*-SNE method was used to present the distribution of the three types of NK cells analyzed (CD56^bri^, CD56^dim^, and CD56^neg^). Each dot corresponds to a single cell, which is colored according to the cell type. **(B)** Dotplot showed the expression of some markers in three NK cell subsets. The dot size represented the percentage of the gene expression in the subset of NK cells and the color represented the average expression of the gene. **(C)** The transcriptome similarity among three NK cell subsets was evaluated by the Euclidean distance and visualized *via* heatmap. Each column represented a variable gene among three NK cell subsets, and each row represented one NK cell subset. **(D)** The DDRTree method were used for dimension reduction to display the distribution of cell types and pseudotime along the trajectory. **(E)** The expression of some genes that changed significantly over pseudotime were shown *via* heatmap. The x-axis represented the pseudotime. The color bar represented the levels of gene expressions. These genes were clustered into 5 clusters by the default “ward.D2” method. **(F)** Global transcriptome differences between CD56^neg^ and CD56^dim^ NK cells were evaluated by overrepresentation analysis of up- and down-regulated biological processes. Avg_log2FC = 0.25 and p_val_adj = 0.05 (Mann-Whitney U-test for significant difference testing, Benjamini-Hochberg method for multiple testing) were selected as thresholds for differentially expressed genes (DEGs). **(G)** Violin plots of module scores for each cell across clusters derived from CD56^bri^, CD56^dim^, and CD56^neg^ groups, which are highlighted in different colors. Horizontal lines represent median values. The significance of the differences was determined using Mann-Whitney U-test and labeled accordingly. ****p < 0.0001.

To detect the characteristics of the CD56^neg^ NK cells in HIV-1-infected patients, we collected an scRNA-seq dataset (SRP150325) from the Sequence Read Archive (SRA). The goal of this approach was to analyze the dataset with respect to the three NK cell subsets in peripheral blood from an HIV-1-infected individual based on CD56 surface marker expression (bright, dim, and negative) (28). After removing cells with low quality, a total of 22,226 cells were identified in this dataset. T cells, B cells and monocytes were identified and exclude for further analysis (**Supplementary Figures 1A–C**). We obtained 20,140 NK cells, including 2,820 CD56^bri^, 6,154 CD56^dim^, and 11,166 CD56^neg^ cells (**Figure 2A**). Some markers of three NK cell subsets were discovered (**Figure 2B**). *GZMK* and *XCL1* were well-defined markers of CD56^bri^ NK cells, *SPON2* and *ALOX5AP* were highly expressed in CD56^dim^ NK cells, while *TRAC* and *ITM2A* were highly expressed in CD56^neg^ NK cells.

The gene expression among three NK cell subsets were further explored. We compared the average expression of all genes from cells in each subset. Euclidean distances analysis reveals that CD56^neg^ NK cells were transcriptionally similar to CD56^dim^ NK cells (**Figure 2C**). These observations were consistent with the result obtained in a previous study showing that CD56^neg^ NK cells were similar to CD56^dim^ NK cells, but only to a certain extent (32). To further investigate the features of CD56^neg^ NK cells compared with the other two NK cell subsets, we first identified those genes differentially expressed between the three types of NK cells (**Supplementary Figure 2A**). We obtained 391, 459, and 331 downregulated DEGs in the “neg vs dim,” “neg vs bri,” and “dim vs bri” groups, respectively. Similarly, we also separately obtained 162, 422, and 636 upregulated DEGs, respectively. Then, pseudotime analysis was used to investigate the developmental course of NK cells (**Figure 2D**). As shown in the trajectory, the CD56^bri^ and CD56^neg^ NK cells dominated the two ends of the progression trajectory. The CD56^dim^ cells dominated another end of the progression trajectory but some cells also distributed in all the three branches (**Supplementary Figure 3**). Based on the current human NK development model, we assigned the CD56^bri^ cells as the least mature branch in the pseudotime. The CD56^bri^, CD56^dim^ and CD56^neg^ cells emerged in turn as the pseudotime. This result provided evidence from transcriptional profiling supporting CD56^dim^ NK cells as the precursors of CD56^neg^ NK cells. The change processes of the 100 most significantly changed genes were displayed along the pseudotime (**Figure 2E**). These genes were clustered into 5 clusters and each cluster of genes displayed similar changing tendency.

Finally, we focused on the functional changes among the three NK cell subsets, especially between CD56^dim^ and CD56^neg^ cells. The 391 downregulated and 162 upregulated DEGs between CD56^neg^ and CD56^dim^ NK cells were displayed (**Supplementary Figure 2B**) and GO enrichment analysis of biological process was performed (**Figure 2F** and **Supplementary Table 4**). GO terms associated with protein synthesis (“SRP-dependent cotranslational protein targeting to membrane”, “protein targeting to ER”, “cotranslational protein targeting to membrane” and “establishment of protein localization to endoplasmic”) were upregulated in CD56^neg^ compared with CD56^dim^ NK cells. In contrast, GO terms including “response to IFN-γ” and “cellular response to IFN-γ” were downregulated. Then, we used function module scores to evaluate functions in each NK cell subset, such as “cytotoxicity”, “cytokine and chemokine receptors”, “adhesion molecules”, “exhaustion”, and “apoptosis” (**Figure 2G**). Compared with CD56^dim^ NK cells, CD56^neg^ NK cells showed a lower cytotoxicity level. The module scores of “cytokine and chemokine receptors” and “adhesion molecules” decreased gradually in the order of CD56^bri^, CD56^dim^, and CD56^neg^ NK cells. However, the “exhaustion” module scores increased in the same order as the NK cell subtypes. Accordingly, “apoptosis” module scores of CD56^neg^ NK cells were also higher than those of CD56^bri^ and CD56^dim^ NK cells.

### Phenotypic Profiles of CD56^neg^ NK Cells in TNs

**Figure 3:**
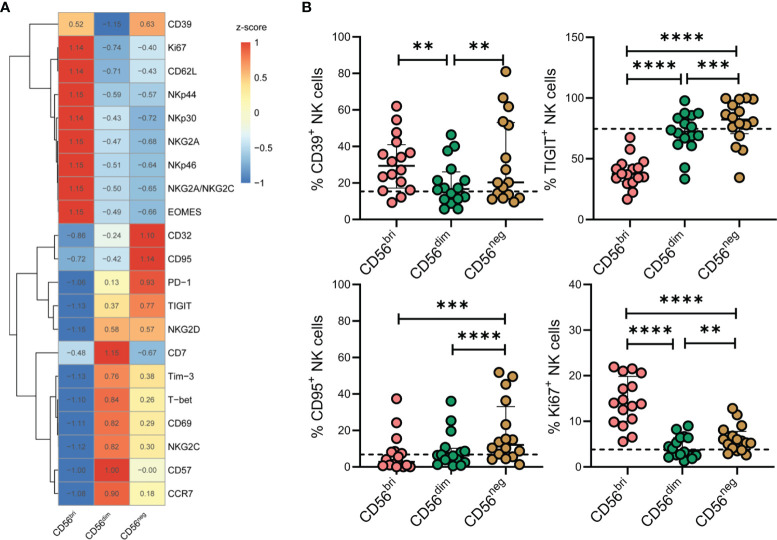
Characteristics of CD56^neg^ NK cells in peripheral blood of treatment-naïve HIV-1-infected patients (TNs) according to flow cytometry analyses. **(A)** Heatmap showing the percentage of cells with specific protein expression across three NK cell subsets, which was determined by flow cytometry. Data were scaled using z-score. **(B)** Expression of CD39, TIGIT, CD95, and Ki67 in NK cell subsets was determined by flow cytometry (n = 16). Dashed lines represent the median expression level of the proteins indicated with respect to the total NK cell population. Each dot represents one participant. For statistical analyses, Wilcoxon signed-rank tests were performed. **p < 0.01, ***p < 0.001, and ****p < 0.0001.

To characterize the phenotypic features of the accumulating CD56^neg^ NK cells in more detail, we measured the expression of a series of markers on CD56^bri^, CD56^dim^, and CD56^neg^ NK cells from TNs by flow cytometry (**Figure 3A**). We found that CD56^neg^ NK cells expressed higher levels of CD39, TIGIT, CD95, and Ki67 compared to CD56^dim^ NK cells (**Figure 3B**). In line with these results, higher levels of transcripts encoding these four proteins were observed in the corresponding scRNA-seq dataset (**Supplementary Figure 4**). In addition, we also compared the expression of these makers on CD56^neg^ NK cells among HCs, TNs and ARTs. We found that CD56^neg^ NK cells in TNs expressed higher levels of CD39, TIGIT, CD95, PD-1, NKG2C, and CD32 compared to that in HCs (**Supplementary Figure 5**).

### CD56^neg^ NK Cell Dysfunction Is Associated With HIV-1 Disease Progression

**Figure 4:**
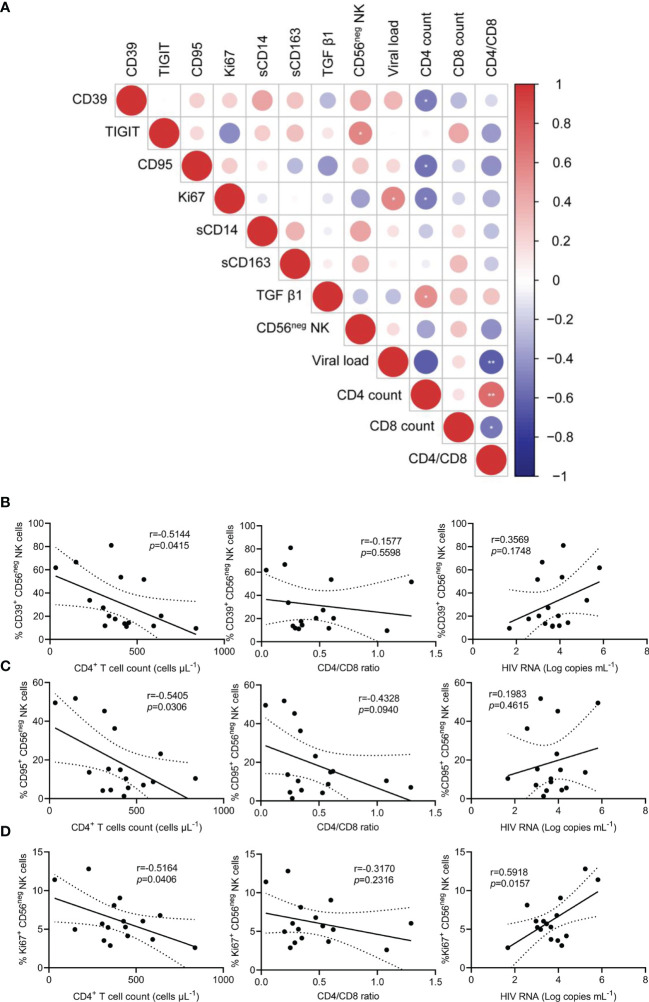
The relationship between the frequencies of CD39^+^, CD95^+^, and Ki67^+^ CD56^neg^ NK cells and peripheral CD4^+^ T cell count, CD4/CD8 ratio, and viral load in treatment-naïve HIV-1-infected patients (TNs). **(A)** Dot heatmap showing Pearson correlations between selected proteins and clinical indicators. **(B–D)** Correlations between the frequencies of CD39^+^ **(B)**, CD95^+^ **(C)**, and Ki67^+^ CD56^neg^ NK cells, **(D)** with CD4^+^ T cell count, CD4/CD8 ratio, and viral load in TNs. Each dot represents one participant. Associations were evaluated using Pearson’s rank correlation test. *p < 0.05 and **p < 0.01.

Chronic inflammation and immune activation play a central role in the progression of HIV-1 infection. To explore the level of inflammation in HIV-1-infected patients, we measured the plasma concentrations of sCD14, sCD163, and TGF-β1. Compared with HCs, the concentrations of the three inflammatory molecules were significantly higher in TNs (**Supplementary Figure 6**). Subsequently, an association of differentially expressed markers on CD56^neg^ NK cells and HIV-1 clinical parameters, including CD4^+^ T cell count, CD4/CD8 ratio, and HIV-1 viral load, was analyzed. Statistical correlations were analyzed using matched measurements presented in a dot heatmap (**Figure 4A**). The frequencies of CD39^+^, CD95^+^, and Ki67^+^ CD56^neg^ NK cells showed significant negative correlations with CD4^+^ T cell counts (**Figures 4B–D**, r = 0.5144, *p* = 0.0415; r = 0.5405, *p* = 0.0306; and r = 0.5164, *p* = 0.0406, respectively). In addition, a positive correlation was observed between the frequencies of Ki67^+^ CD56^neg^ NK cells and HIV-1 viral load (**Figure 4D**). These data highlight that immune dysregulation of CD56^neg^ NK cells is associated with HIV-1 disease progression.

### Functional Impairment of CD56^neg^ NK Cells in TNs

**Figure 5:**
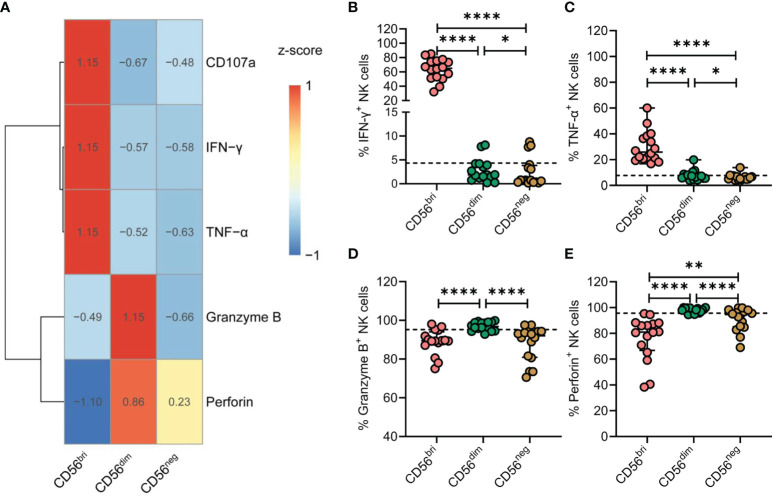
CD56^neg^ NK cells derived from treatment-naïve HIV-1-infected patients (TNs) display impaired immune functions. **(A)** Heatmap showing the percentage of cells featuring specific gene expression patterns across the three subtypes of NK cells. Data were scaled using z-score. **(B–E)** Peripheral blood mononuclear cells (PBMCs) from TNs were stimulated with IL-12 (10 ng/mL), IL-15 (10 ng/mL), and IL-18 (50 ng/mL) for 20 h. Comparisons between CD56^bri^, CD56^dim^, and CD56^neg^ NK cells regarding the frequencies of cells producing IFN-γ **(B)** and TNF-α **(C)**, and those expressing granzyme B **(D)** and perforin **(E)**. Dashed lines indicate the median levels of makers with respect to the total NK cell population. Each dot represents one participant. For statistical analyses, Wilcoxon signed-rank tests were performed. *p < 0.05, **p < 0.01, and ****p < 0.0001.

NK cells are best characterized by their cytotoxic functions and their ability to produce cytokines. Thus, NK cells from TNs were stimulated by treatment with IL-12, IL-15, and IL-18. Expression of CD107a, IFN-γ, TNF-α, granzyme B, and perforin was subsequently determined by flow cytometry (**Figure 5A**). Compared with CD56^dim^ NK cells, the expression of IFN-γ and TNF-α was significantly decreased in CD56^neg^ NK cells (*p* = 0.0214 and *p* = 0.0207, respectively; **Figures 5B, C**). In addition, CD56^neg^ NK cells expressed lower levels of granzyme B and perforin than CD56^dim^ NK cells (*p* < 0.0001 and *p* < 0.0001, respectively; **Figures 5D, E**). Moreover, we compared the expression of the four genes of interest between the three subtypes of NK cells at the transcriptional level based on the scRNA-seq dataset (**Supplementary Figure 7**). The transcript levels of *GZMB* and *PRF1* genes analyzed corresponded to the respective protein levels in the three NK cell subtypes. Simultaneously, we found the expression of TNF-α of CD56^neg^ NK cells was significantly decreased in TNs and ARTs compared to HCs (**Supplementary Figure 8**).

Taken together, these data indicate that chronic HIV-1 infection drives the expansion of CD56^neg^ NK cells that express lower levels of granzyme B and perforin and display defective production of cytokines, such as IFN-γ and TNF-α.

## Discussion

The NK cell compartment is heterogeneous in nature and contains a variety of subsets with different maturities, phenotypes, and functions. One factor that is known to drive this heterogeneity is viral infection (22, 33). HIV-1 infection has been associated with severe disruption of the NK cell compartment (34). HIV-1 affects the homeostasis of NK cell subsets by inducing a reduction in CD56^dim^ NK cells and an accumulation of CD56^neg^ NK cells (14, 21). However, the immunological characteristics of CD56^neg^ NK cell populations are still poorly understood. In this study, we uncovered an association between the accumulation of dysfunctional CD56^neg^ NK cells and disease progression in chronic HIV-1 infection.

Previous studies have indicated decreases in both frequency and absolute numbers of CD56^dim^ NK cells, with a concomitant increase in the CD56^neg^ NK cell population in HIV-1 infection (21, 35). Consistently, in our study, we confirmed the expansion of the CD56^neg^ NK cell population. Similarly, expansion of CD56^neg^ NK cells has also been observed during infections with other viruses, including HCV, cytomegalovirus, Epstein–Barr virus, and HBV (36–39). More recently, unconventional CD56^neg^ NK cells were shown to result from aberrant maturation of conventional NK cells, and a high frequency of CD56^neg^ NK cells was found to be associated with adverse clinical outcomes in acute lymphoblastic leukemia (40). To our knowledge, an in-depth study in the context of HIV-1 infection has not been undertaken to this point.

CD56^neg^ NK cells have been reported to be similar to CD56^dim^ NK cells in healthy individuals but with phenotypic differences in CD56^neg^ NK cells (32). In this study, we collected an scRNA-seq dataset obtained using NK cells in the peripheral blood of an HIV-1-infected individual from SRA. Consistent with proteomics data, we also demonstrated that CD56^neg^ NK cells were closer to CD56^dim^ NK cells than CD56^bri^ NK cells according to the Euclidean distances and pesudotime analysis among three NK cell subsets. Module score analysis showed that CD56^neg^ NK cells exhibited lower cytotoxicity and adhesion, but higher levels of exhaustion and apoptosis, suggesting that these CD56^neg^ NK cells were dysfunctional. By employing flow cytometry, we observed that CD56^neg^ NK cells from patients infected with HIV-1 exhibited a lower production capacity for IFN-γ and TNF-α, as well as reduced cytotoxic functions in response to stimulation by IL-12, IL-15, and IL-18 compared to CD56^dim^ NK cells. During our enrichment analysis, we found that multiple GO terms related to cell activation were decreased, including “T cell activation” and “neutrophil activation” which suggested that viral infection may also significantly impair CD56^neg^ cell functions. We observed that the expression of two genes, including killer cell lectin-like receptor subfamily C member 1 (KLRC1) 1 and killer cell lectin-like receptor subfamily D member 1 (KLRD1) were downregulated in CD56^neg^ NK cells. KLRC1 has been proposed to function as an immune inhibitory receptor involved in self–nonself discrimination. Complexes of KLRC1 and KLRD1 enable cytotoxic cells to monitor the expression of major histocompatibility complex (MHC) class I molecules in healthy cells to mediate self-tolerance (41–43). Deregulation of these genes may be responsible for the increased levels of exhaustion and apoptosis and the decrease in cytotoxicity observed in CD56^neg^ NK cells.

NK cell function relies on the expression patterns of activating and inhibitory receptors. Phenotypic characterization of CD56^neg^ NK cells revealed a loss of certain activating receptors, such as the natural cytotoxicity receptors NKp30 and NKp46, as well as decreased expression of NKG2A (14, 44). Given that NK cell activation relies on an integration of signals from activating and inhibitory receptors, the alteration of NK cell receptor expression may indirectly influence the CD56^neg^ NK cell response in HIV-1-infected patients. CD39, a member of the ecto-nucleoside triphosphate diphosphohydrolase family, converts ATP and ADP to AMP, which is a newly recognized “immune checkpoint mediator.” TIGIT, an inhibitory receptor, binds to its ligand CD155 to generate inhibitory signals. Cell surface expression of TIGIT is higher on NK cells from patients infected with HIV-1 than on those from HIV-1-negative HCs (18). In our study, we demonstrated that the expression of CD39 and TIGIT was higher on CD56^neg^ than on CD56^dim^ NK cells. Notably, the frequencies of CD39 and TIGIT on CD56^neg^ NK cells correlated negatively with the absolute number of CD4^+^ T cells, which is an important indicator of HIV-1 disease progression. Moreover, we also found that CD95 and Ki67 expression levels were higher in CD56^neg^ than in CD56^dim^ NK cells. This indicated that both proliferation and apoptosis of CD56^neg^ NK cells were increased, which ultimately led to the observed accumulation of CD56^neg^ NK cells. Furthermore, the expression of CD39 and TIGIT on CD56^neg^ NK cells correlated negatively with the absolute number of CD4^+^ T cells. Although further mechanisms of dysregulation of CD56^neg^ NK cell functions need to be addressed in the future, this is the first study to demonstrate the relationship between the phenotypic profile of CD56^neg^ NK cells and HIV-1 disease progression.

We acknowledge the limitations of our study. First, since this was a cross-sectional study, the causal relationship between the accumulation of CD56^neg^ NK cells and disease progression is not yet known. It is thus suggested to carry out further studies in a longitudinal ART cohort. Secondly, scRNA-seq data from only one HIV-1 infected patient with bnAb production is included. Future studies should aim to ascertain whether the gene expression pattern of CD56^bri^, CD56^dim^ and CD56^neg^ NK cells characterized here are similar to those in HIV-infected patients without bnAb production. In addition, the main conclusions of this study were based on observations made using PBMCs. Considering that HIV-1 persists mainly in tissues, additional attention should be paid to the function and distribution of CD56^neg^ NK cells in tissues.

In conclusion, our study demonstrated the impact of chronic HIV-1 infection on the immune characteristics of CD56^neg^ NK cells and revealed that immune dysfunctions of CD56^neg^ NK cells are associated with HIV-1 disease progression. Thus, exploration of the mechanisms involved in the expansion of CD56^neg^ NK cells could prove helpful in the development of novel immunotherapies to restore or reinvigorate dysfunctional NK cells during chronic HIV-1 infection.

## Data Availability Statement

The datasets presented in this study can be found in online repositories. The names of the repository/repositories and accession number(s) can be found in the article/**Supplementary Material**.

## Ethics Statement

The studies involving human participants were reviewed and approved by the Fifth Medical Center of Chinese PLA General Hospital Research Ethics Committee. The patients/participants provided their written informed consent to participate in this study.

## Author Contributions

S-QW, F-SW, and J-WS conceived, designed, and supervised experiments. W-JC and L-YW collected clinical samples and performed the experiments. L-YW and L-LS collected clinical information. C-BZ and J-HY performed flow cytometry. W-JC and X-CZ performed the statistical analysis and figures. J-WS, W-JC, X-CZ and Q-YL wrote the manuscript. X-YM, A-LG, M-JZ, CZ, XF, Y-MJ, R-NX, S-QW, F-SW, and J-WS provided comments and feedback. All authors edited and approved the final manuscript.

## Funding

This work was supported by the National Science and Technology Major Project (2018ZX10302104-002-001), National Natural Science Foundation of China (82101837, 81772185, 81830101) the Innovative Research Team in the National Natural Science Foundation of China (81721002), and Science and Technology Key Research & Development Program of Nanning (20193008).

## Conflict of Interest

The authors declare that the research was conducted in the absence of any commercial or financial relationships that could be construed as a potential conflict of interest.

## Publisher’s Note

All claims expressed in this article are solely those of the authors and do not necessarily represent those of their affiliated organizations, or those of the publisher, the editors and the reviewers. Any product that may be evaluated in this article, or claim that may be made by its manufacturer, is not guaranteed or endorsed by the publisher.
