# Supplementary material for: Immune Dysfunctions of CD56neg NK Cells Are Associated With HIV-1 Disease Progression
Source: Front Immunol. 2022 Jan 7;12:811091. doi: 10.3389/fimmu.2021.811091 (PMC8777256; doi:10.3389/fimmu.2021.811091)
Supplement: Supplementary file 1 [file DataSheet_1.pdf]

Supplementary Information

Immune dysfunctions of CD56^{neg} NK cells are associated with HIV-1 disease progression

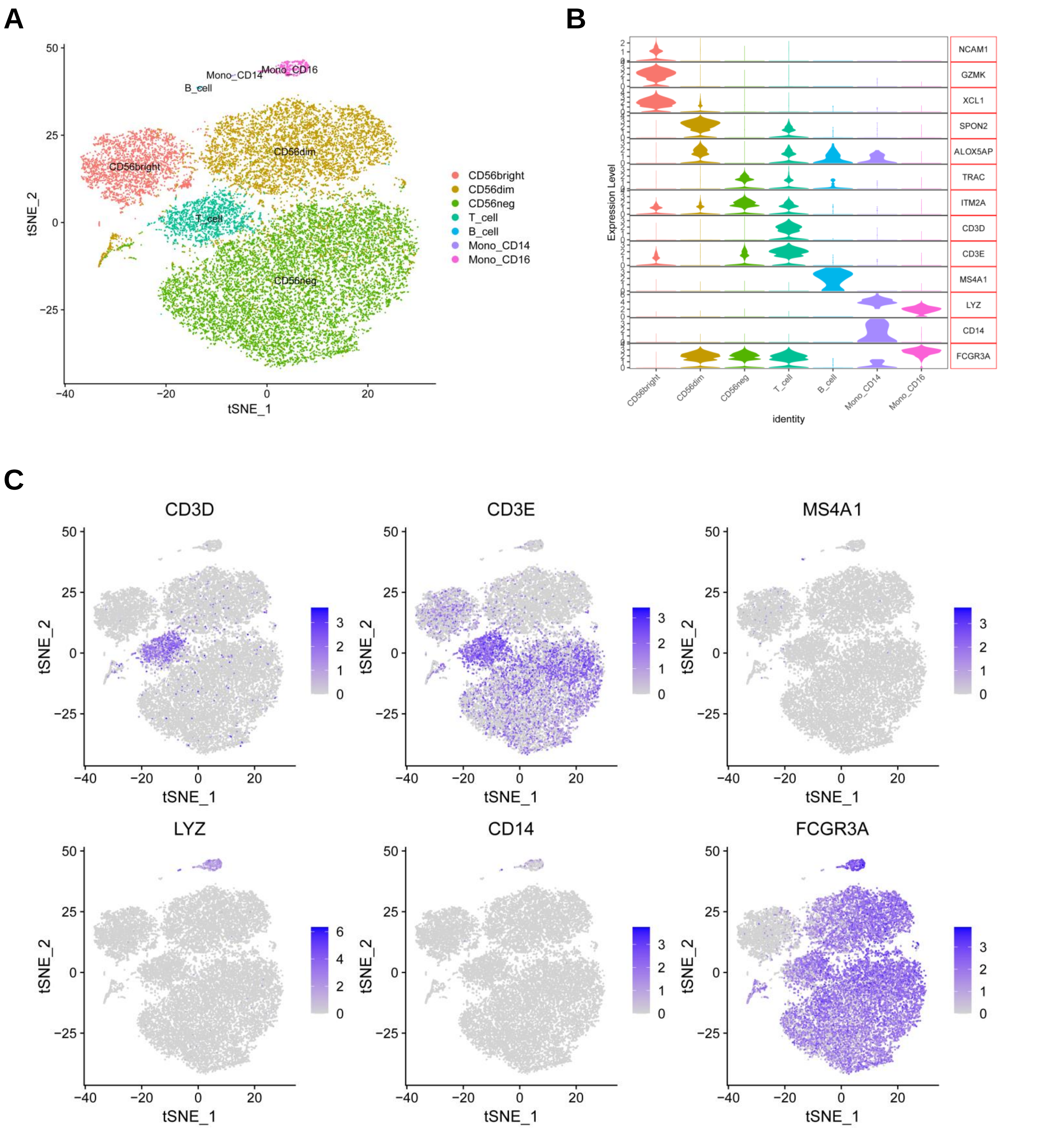

Supplementary Figure 1. Excluding the cells that did not belong to NK cells

(A) TSNE dimplot showed the annotation of all the 7 types of cells in the dataset. **(B)** Vlnplot showed the expressions of canonical markers of the 7 types of cells. **(C)** The expression of canonical markers of T cells, B cells, and monocyte (Mono CD14/CD16) were shown.

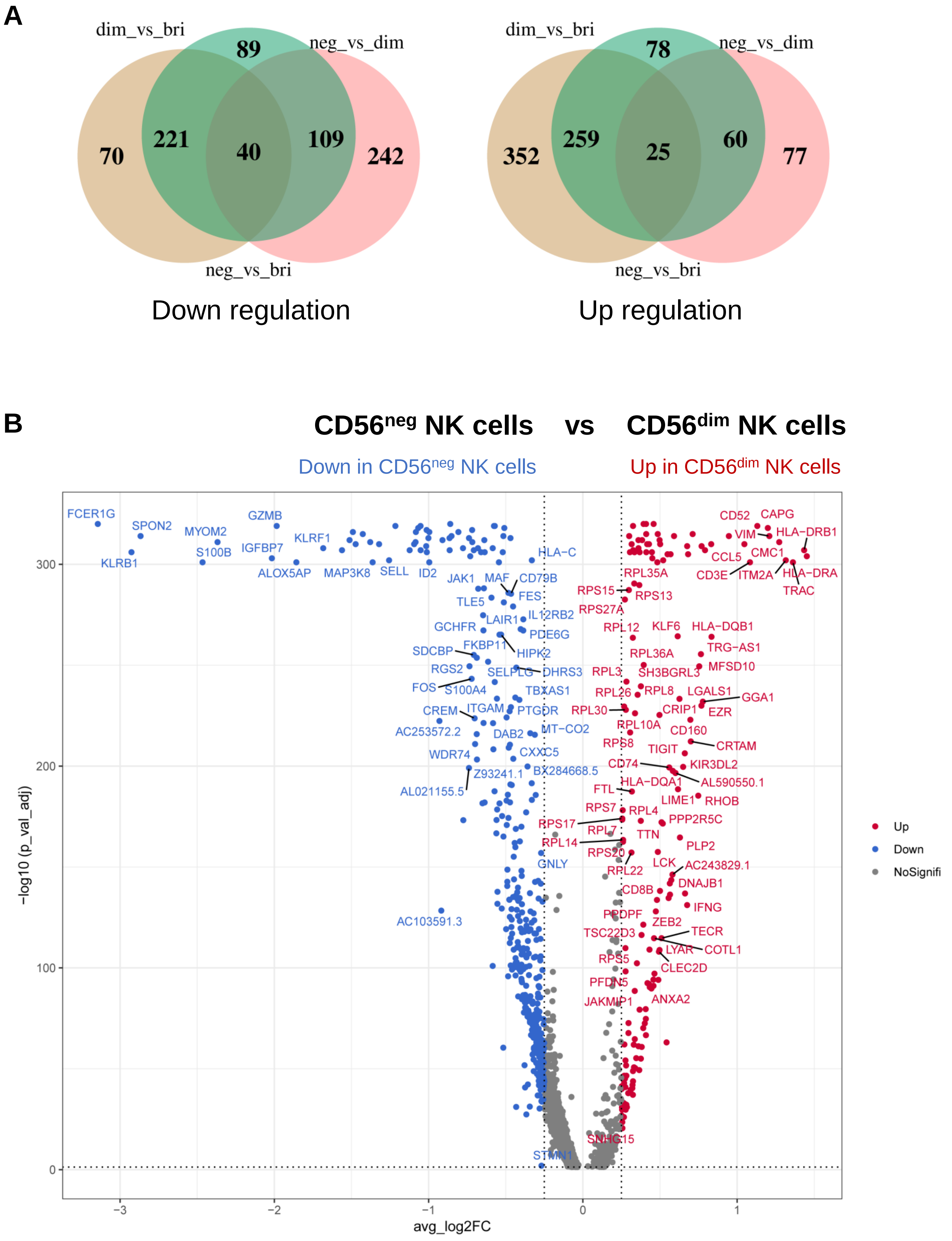
A
dim_vs_bri
neg_vs_dim
neg_vs_bri
89
70
221
40
109
242
Down regulation
dim_vs_bri
neg_vs_dim
neg_vs_bri
78
352
259
25
60
77
Up regulation
B
CD56neg NK cells vs CD56dim NK cells
Down in CD56neg NK cells
Up in CD56dim NK cells
FCER1G
SPON2
MYOM2
GZMB
KLRF1
IGFBP7
S100B
KLRB1
ALOX5AP
MAP3K8
SELL
ID2
HLA-C
JAK1
MAF
CD79B
TLE5
FES
LAIR1
IL12RB2
GCHFR
PDE6G
FKBP11
HIPK2
SDCBP
RGS2
SELPLG
DHRS3
FOS
S100A4
TBXAS1
CREM
ITGAM
PTGDR
AC253572.2
DAB2
MT-CO2
WDR74
CXXC5
Z93241.1
BX284668.5
AL021155.5
GNLY
AC103591.3
STMN1
CD52
CAPG
VIM
HLA-DRB1
CCL5
CMC1
CD3E
ITM2A
HLA-DRA
TRAC
RPL35A
RPS15
RPS13
RPS27A
RPL12
KLF6
HLA-DQB1
TRG-AS1
RPL36A
MFSD10
RPL3
SH3BGRL3
RPL26
RPL8
LGALS1
GGA1
RPL30
CRIP1
EZR
RPL10A
CD160
RPS8
TIGIT
CRTAM
CD74
KIR3DL2
HLA-DQA1
AL590550.1
FTL
LIME1
RHOB
RPS7
RPL4
PPP2R5C
RPS17
RPL7
TTN
RPL14
PLP2
RPS20
LCK
AC243829.1
RPL22
DNAJB1
CD8B
IFNG
PFDPF
ZEB2
TSC22D3
TECR
LYAR
COTL1
RPS5
CLEC2D
PFDN5
JAKMIP1
ANXA2
SNHG15
300
200
100
0
−log10 (p_val_adj)
−3
−2
−1
0
1
avg_log2FC
Up
Down
NoSignifi

Supplementary Figure 2. Differentially expressed genes (DEGs) in the three NK cell subsets

(A) Two Venn diagrams separately showing the relationship of up- and downregulated DEGs between three NK cell subsets. These DEGs were identified using a threshold of "avg_log2FC > 0.25 & p_val_adj < 0.05". **(B)** Volcano plot showing a more detailed analysis of up- and downregulated DEGs between $CD56^{neg}$ and $CD56^{dim}$ NK cells.

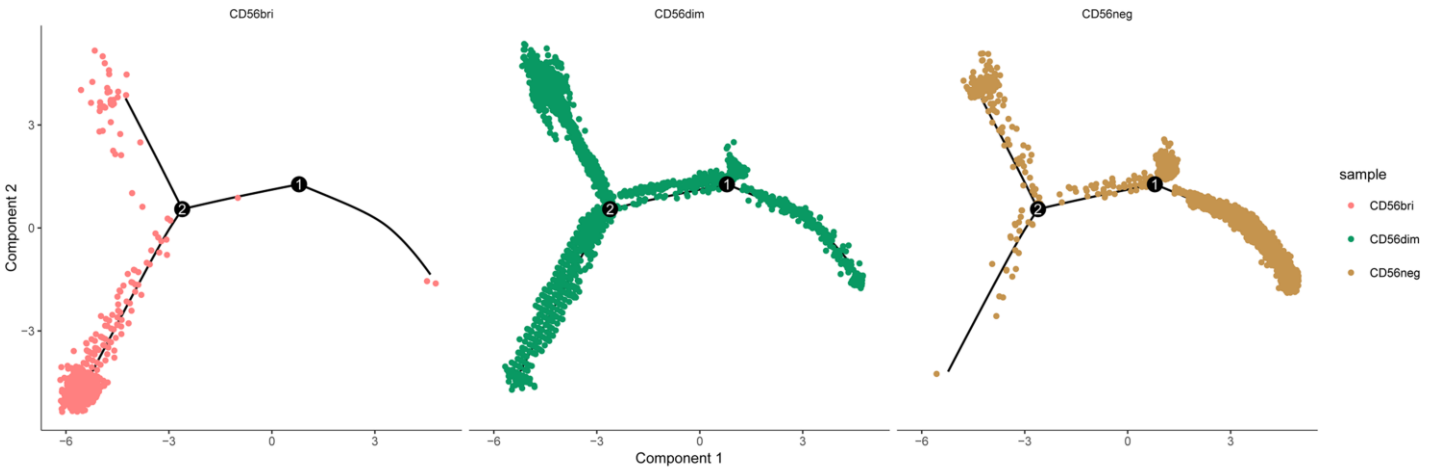

Supplementary Figure 3. The distributions of three NK cell subsets in pseudotime analysis

Three dotplots showing the distributions of three NK cell subsets separately. The DDRTree algorithm was used for dimension reduction.

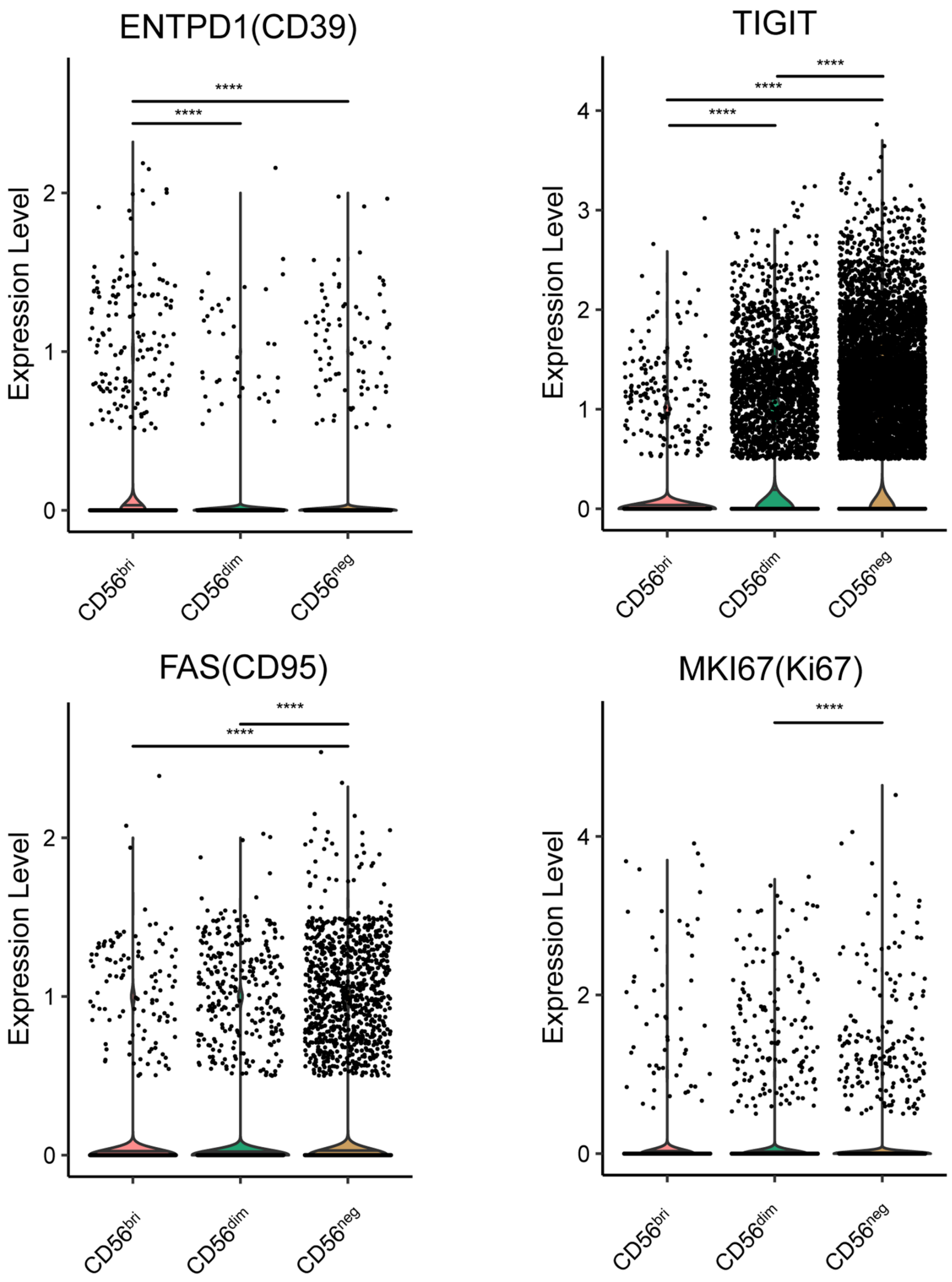

Supplementary Figure 4. Jitter and violin plots showing single-cell expression levels of selected genes in cells derived from peripheral blood of HIV-1-infected patients

Expression levels were calculated by $log_2(count + 1)$, in which $count$ represents the unique molecular identifier (UMI) count of a particular gene. Statistical significance between two groups was calculated by Mann-Whitney U-test. *p < 0.05, **p < 0.01, and ****p < 0.0001

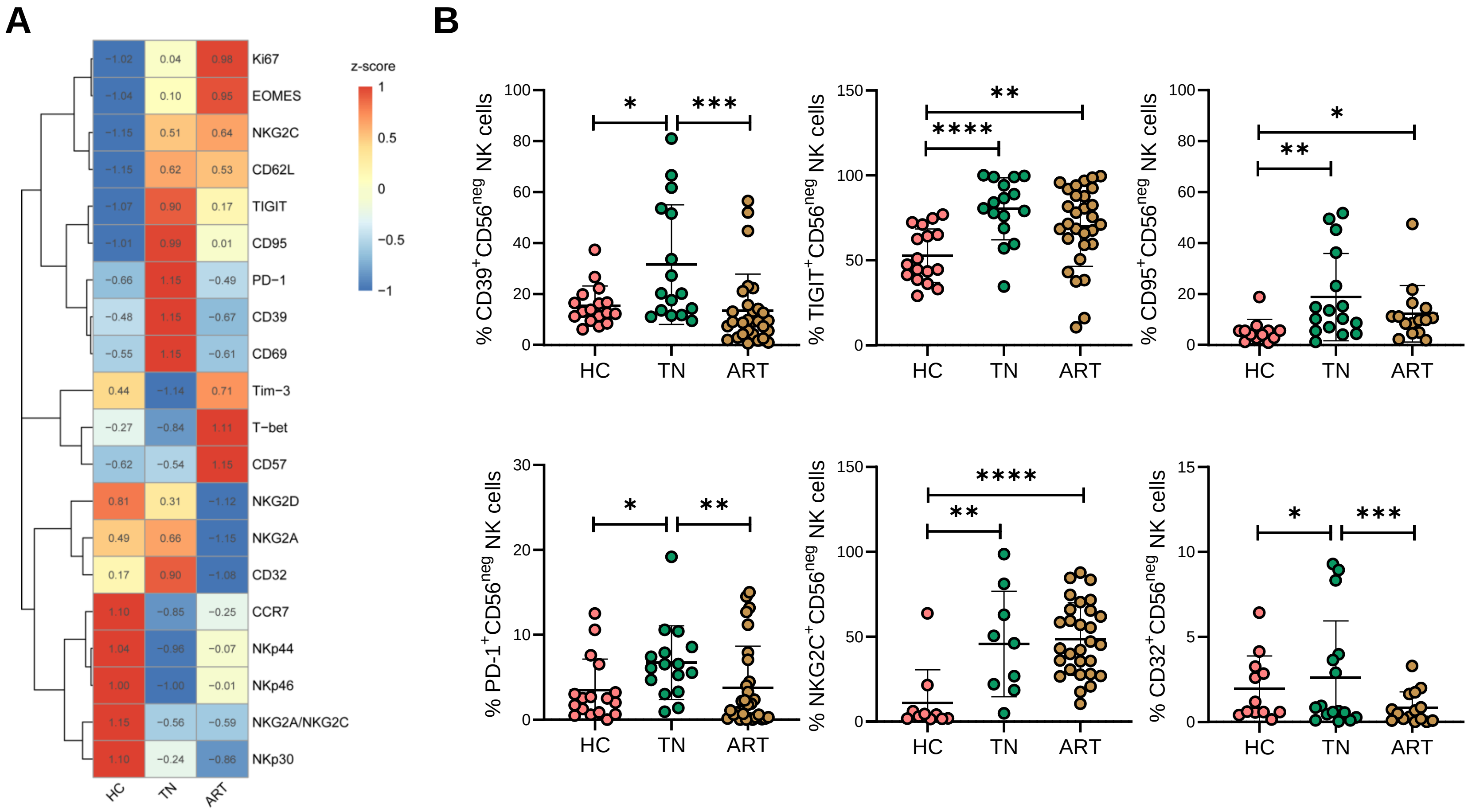

Supplementary Figure 5. Phenotypic of CD56^{neg} NK cells among three groups of participants

(A) Heatmap showing the percentage of CD56^{neg} NK cells with specific protein expression across the three groups of participants, which was determined by flow cytometry. Data were scaled using z-score. **(B)** Expression of CD39, TIGIT, CD95, PD-1, NKG2C, and CD32 of CD56^{neg} NK cells in three groups was determined by flow cytometry. Each dot represents one participant. Statistical significance between two groups was determined by Mann-Whitney U-test. **p* < 0.05, ***p* < 0.01, ****p* < 0.001, and *****p* < 0.0001

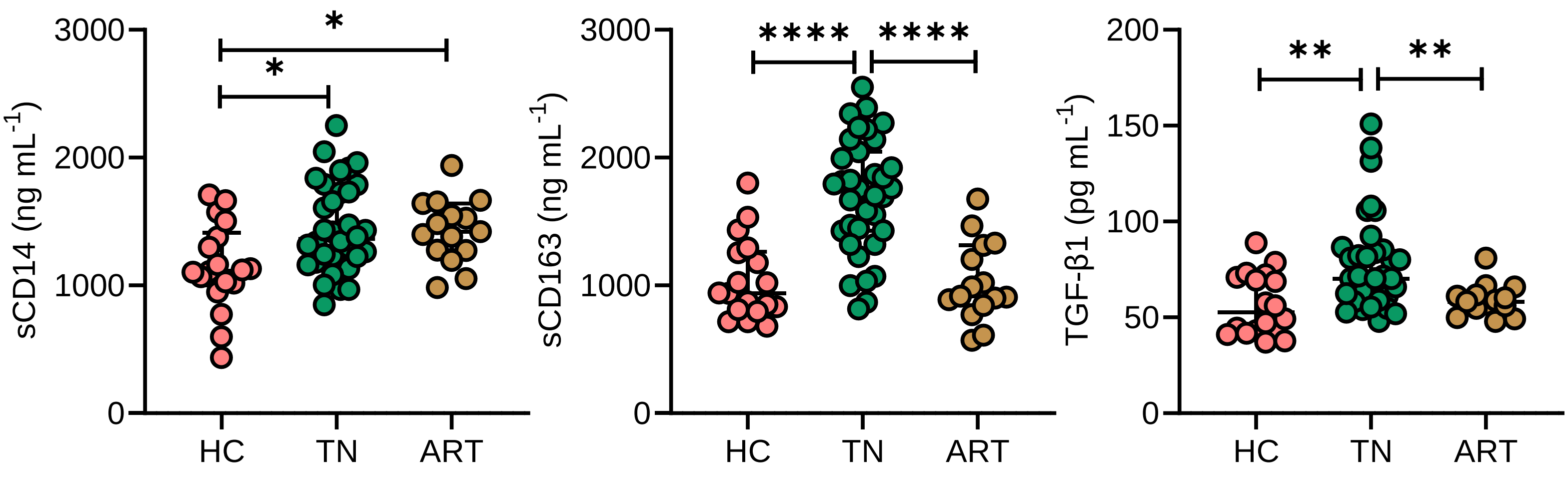

Supplementary Figure 6. The level of plasma inflammatory markers in healthy controls and HIV-1-infected participants

Comparisons of the plasma levels of sCD14, sCD163, and TGF-β1 in HCs (n = 18), treatment-naïve HIV-1-infected patients (TNs; n = 34), and HIV-1-infected patients with successful antiretroviral therapy (ARTs; n = 15). Each dot represents one participant. Statistical significance between two groups was determined using Mann-Whitney U-test. $*p < 0.05$, $**p < 0.01$, and $****p < 0.0001$

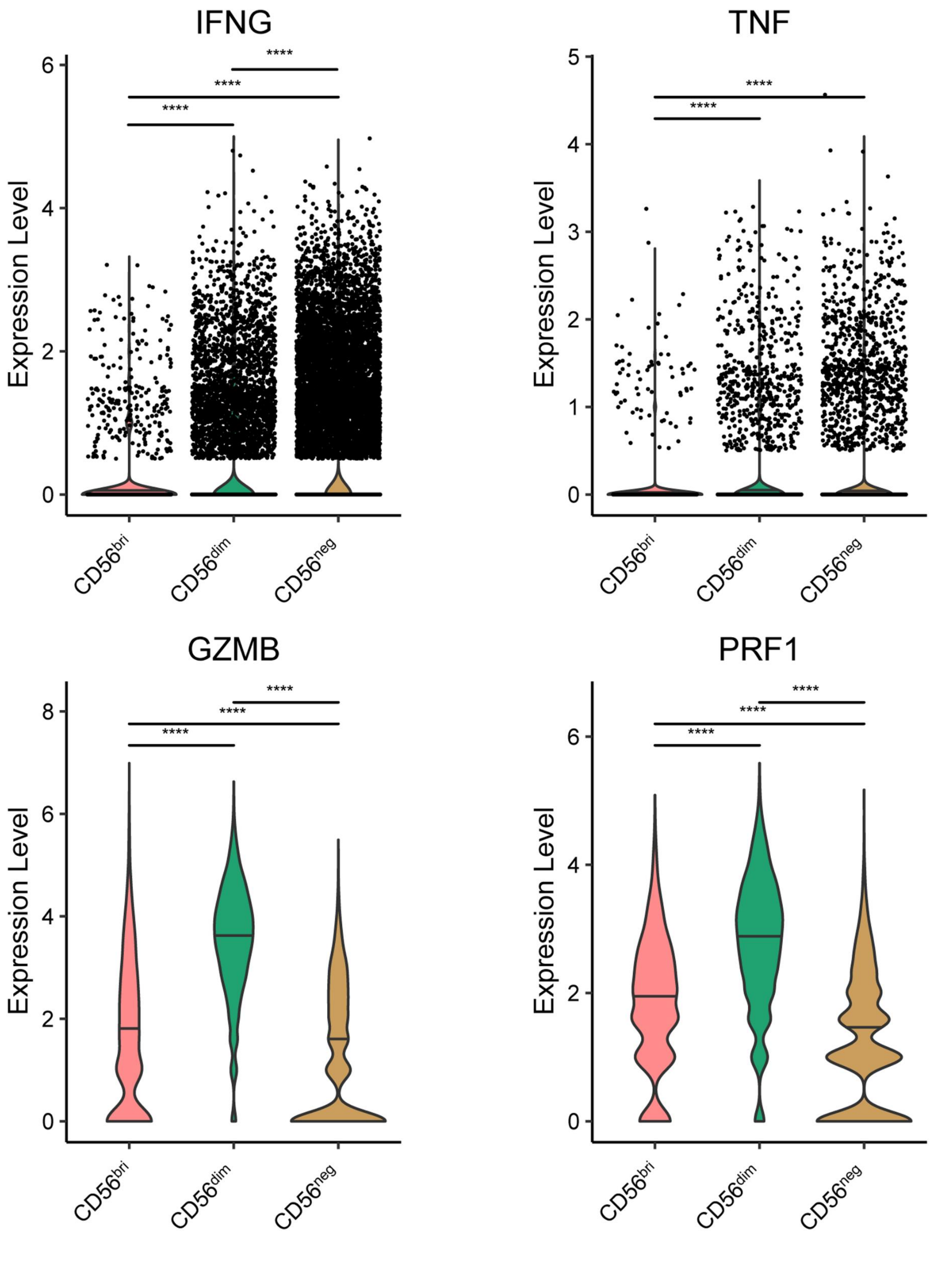

Supplementary Figure 7. Jitter and violin plots showing single-cell expression levels of selected genes in cells derived from peripheral blood of HIV-1-infected patients

Expression levels were calculated by $log_2(count + 1)$, in which $count$ represents the unique molecular identifier (UMI) count of a particular gene. Statistical significance between two groups was calculated using Mann-Whitney U-test. ****p < 0.0001

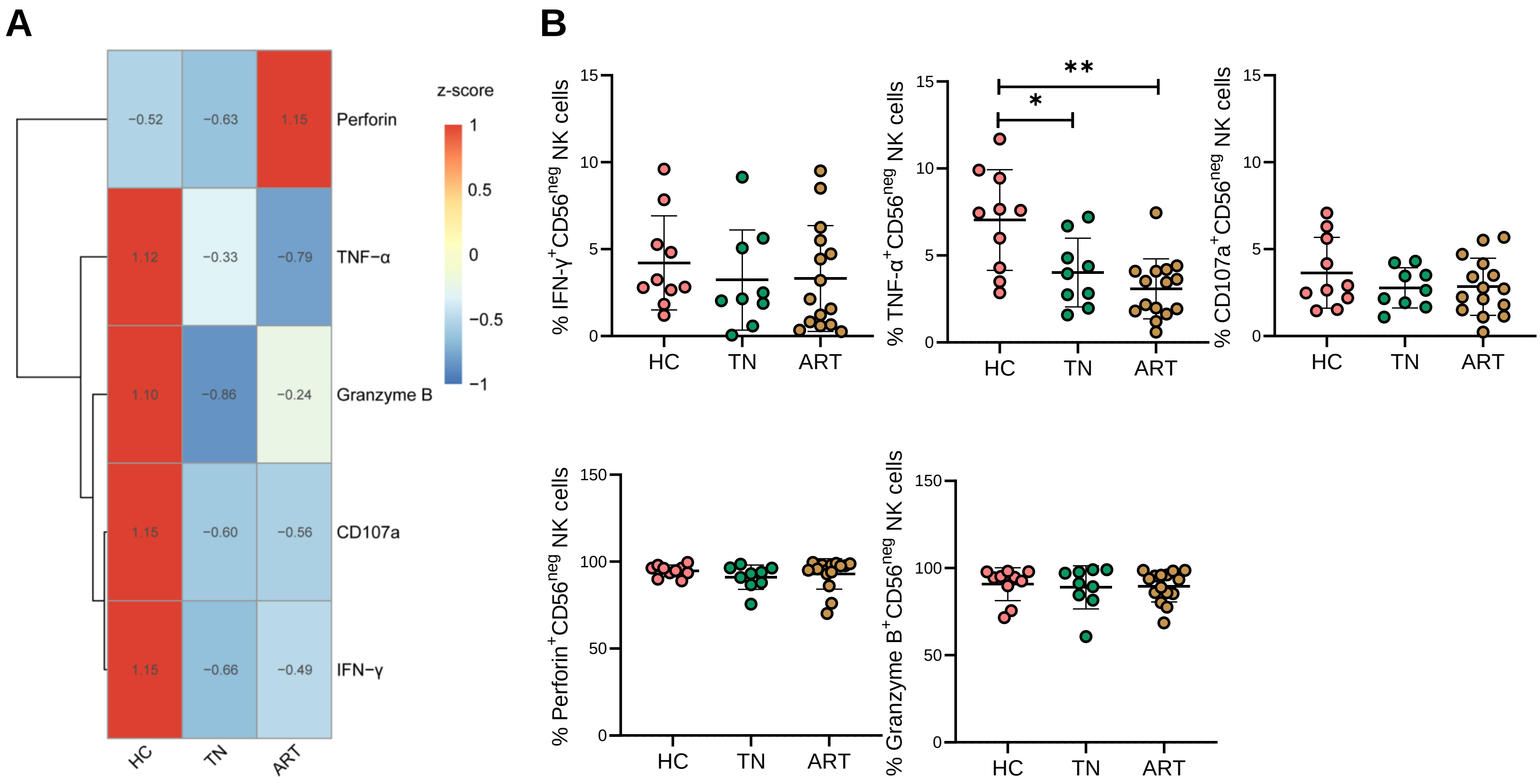

Supplementary Figure 8. The functions of CD56neg NK cells from three groups of participants

Heatmap **(A)** and scatter plot **(B)** showing the expression of IFN-γ, TNF-α, CD107a, perforin, and granzyme B in CD56neg NK cells from HC, TN, ART. Each dot represents one participant. Statistical significance between two groups was determined by Mann-Whitney U-test. *p < 0.05 and **p < 0.01

Supplementary Table 1. Characteristics of HIV-1-infected patients with successful antiretroviral therapy (ARTs)

**Characteristics**	**ARTs (n=29)**
CD4 Count before ART, cells/μL, Median (IQR)	382 (320~445)
Viral load before ART, Log copies/mL, median (IQR)	4.54 (4.06~4.83)
Duration of ART, months, median (IQR)	38 (26~43)
ART regimen, n (%)	
3TC/TDF/EFV	27 (93)
3TC/AZT/EFV	2 (7)
Transmission route, n (%)	
MSM	26 (90)
Heterosexual	2 (7)
Blood transfusion	1 (3)

All values, except for the ART regimen and transmission route, are expressed as median (interquartile range, IQR). Abbreviations: 3TC, lamivudine; TDF, tenofovir disoproxil fumarate; EFV, efavirenz; AZT, zidovudine; MSM, men who have sex with men

Supplementary Table 2. Reagents used for flow cytometry analyses

Antibodies	Source	Clone	Identifier
APC-Fire 750 anti-human CD3	BioLegend	SK7	344840
APC-Fire 750 anti-human CD14	BioLegend	63D3	367120
APC-Fire 750 anti-human CD20	BioLegend	2H7	302358
PerCP anti-human CD16	BioLegend	3G8	302030
BV421 anti-human CD16	BD	3G8	562874
BV421 anti-human CD56	BioLegend	HCD56	318328
APC anti-human CD56	Tongshengshidai	NA	Z6410017
PE-Cy7 anti-human CD56	BioLegend	HCD56	318318
APC anti-human-CD39	BioLegend	A1	328210
FITC anti-human Ki67	BioLegend	Ki-67	350508
PE-Cy7 anti-human CD62L	BioLegend	DREG-56	304822
AF647 anti-human NKp44	BD	P44-8.1	558563
AF647 anti-human NKp30	BD	P30-15	558408
FITC anti-human NKG2A	MACS	REA110	130-113-565
BV510 anti-human NKp46	BioLegend	9E2	331924
eF660 anti-human EOMES	Thermo Fisher	WD1928	50-4877-42
PE-Cy7 anti-hum Tim-3	Thermo Fisher	F38-2E2	25-3109-42
PE anti-hum Tim-3	BD	7D3	565570
BV421 anti-human T-bet	BioLegend	4B10	644816
BV510 anti-human CD69	BioLegend	FN50	310936
PE-Cy7 anti-human CD69	BioLegend	FN50	557745
PE anti-human NKG2C	BioLegend	S19005E	375004
FITC anti-human CD57	BioLegend	HNK-1	359604
APC anti-human CCR7	BD	G043H7	353214
FITC anti-human CD32	BioLegend	FUN-2	303204
PE anti-human CD95	BD	DX2	555674
BV510 anti-human PD-1	BioLegend	EH12.1	563076
PE anti-human TIGIT	BioLegend	A15153G	372704
PE-Cy7 anti-human NKG2D	BioLegend	1D11	320812
APC anti-human CD7	BioLegend	CD7-6B7	343108
FITC anti-human CD107a	BioLegend	H4A3	328606
FITC anti-human IFN-γ	BioLegend	4SB3	502506
BV421 anti-human TNF-α	BioLegend	MAb11	502932
APC anti-human Granzyme B	BD	GB11	560212
PE-Cy7 anti-human Perforin	BioLegend	dG9	308126

Supplementary Table 3. Selected genes used for calculating five function module scores

Function Modules	Genes Used for Module Score
Cytotoxicity	GZMA, GZMB, GZMH, GZMM, GZMK, GNLY, PRF1, CTSW
Cytokine and Chemokine Receptors	IL7R, IL2RB, IL21R, CXCR3, CXCR4, CCR7, CXCR1, S1PR5
Adhesion Molecules	ITGAL, CD2, CD58, ITGB2, SELL, CD96
Exhaustion	LAG3, TIGIT, PDCD1, HAVCR2, TOX
Apoptosis	FAS, CRIP1, IFI27, CD74, IFNG, HERPUD1, HMGB2, LCK, BCL2A1, RPL11, TYROBP, CD3E, LGALS3, JUN, PRELID1, IFI27L2

Supplementary Table 4. Top 10 upregulated and downregulated biological process

ID	Description	GeneRatio	BgRatio	pvalue	p.adjust	qvalue	Count	Regulation
GO:0006614	SRP-dependent cotranslational protein targeting to membrane	44/147	105/18862	7.93E-67	1.85E-63	1.54E-63	44	Up
GO:0006613	cotranslational protein targeting to membrane	44/147	110/18862	1.06E-65	6.62E-63	5.51E-63	44	Up
GO:0000184	nuclear-transcribed mRNA catabolic process, nonsense-mediated decay	45/147	120/18862	1.14E-65	6.62E-63	5.51E-63	45	Up
GO:0045047	protein targeting to ER	45/147	120/18862	1.14E-65	6.62E-63	5.51E-63	45	Up
GO:0072599	establishment of protein localization to endoplasmic reticulum	45/147	124/18862	6.94E-65	3.23E-62	2.69E-62	45	Up
GO:0070972	protein localization to endoplasmic reticulum	45/147	152/18862	3.80E-60	1.47E-57	1.23E-57	45	Up
GO:0019083	viral transcription	46/147	180/18862	3.67E-58	1.22E-55	1.02E-55	46	Up
GO:0006413	translational initiation	46/147	193/18862	1.35E-56	3.93E-54	3.27E-54	46	Up
GO:0019080	viral gene expression	46/147	198/18862	5.01E-56	1.30E-53	1.08E-53	46	Up
GO:0000956	nuclear-transcribed mRNA catabolic process	45/147	208/18862	3.16E-53	7.37E-51	6.14E-51	45	Up
GO:0034341	response to interferon-gamma	27/341	197/18862	2.49E-16	9.64E-13	7.50E-13	27	Down
GO:0071346	cellular response to interferon-gamma	24/341	177/18862	1.53E-14	2.96E-11	2.30E-11	24	Down
GO:0042110	T cell activation	36/341	474/18862	3.61E-13	4.67E-10	3.63E-10	36	Down
GO:0002831	regulation of response to biotic stimulus	32/341	420/18862	7.33E-12	7.10E-09	5.52E-09	32	Down
GO:0007159	leukocyte cell-cell adhesion	29/341	366/18862	2.88E-11	2.23E-08	1.74E-08	29	Down
GO:0042119	neutrophil activation	34/341	500/18862	3.59E-11	2.32E-08	1.80E-08	34	Down
GO:0002683	negative regulation of immune system process	30/341	403/18862	6.08E-11	3.37E-08	2.62E-08	30	Down
GO:0043312	neutrophil degranulation	32/341	485/18862	3.00E-10	1.46E-07	1.13E-07	32	Down
GO:0002283	neutrophil activation involved in immune response	32/341	488/18862	3.51E-10	1.51E-07	1.18E-07	32	Down
GO:0002446	neutrophil mediated immunity	32/341	499/18862	6.13E-10	2.38E-07	1.85E-07	32	Down
